# Towards the implementation of malaria elimination policy in South Africa: the stakeholders’ perspectives

**DOI:** 10.1080/16549716.2017.1288954

**Published:** 2017-05-05

**Authors:** Khumbulani Welcome Hlongwana, Joyce Tsoka-Gwegweni

**Affiliations:** ^a^ Public Health Medicine, University of KwaZulu-Natal, Durban, South Africa

**Keywords:** Epidemiology, interventions, achievability, researchers, malaria programme

## Abstract

**Background**: The past decade has seen substantial global reduction in malaria morbidity and mortality due to increased international funding and decisive steps by the international malaria community to fight malaria. South Africa has been declared ready to institute malaria elimination. However, research on the factors that would affect this policy implementation is inadequate.

**Objective**: To investigate the stakeholders’ understanding of the malaria elimination policy in South Africa, including their perceived barriers and facilitators to effective policy implementation.

**Methods**: The study followed a constructivist epistemological approach which manifests in phenomenological study design. Twelve purposively selected key informants from malaria researchers, provincial and national malaria programmes were interviewed using semi-structured interviews. Interview questions elicited interviewees’ knowledge of the policy and its achievability, including any perceived barriers and facilitating factors to effective implementation. The hybrid approach was used to perform thematic data analysis.

**Results**: The dominant view was that malaria remains a problem in South Africa, exacerbated by staff attitudes and poor capacity, lack of resources, lack of new effective intervention tools, lack of intra- and inter-departmental collaboration, poor cross-border collaboration and weak stakeholder collaboration. Informants were concerned about the target year (2018) for elimination, and about the process followed in developing the policy, including the perceived malaria epidemiology shortfalls, regulatory issues and political context of the policy.

**Conclusions**: Achievability of malaria elimination remains a subject of intense debate for a variety of reasons. These include the sporadic nature of malaria resurgence, raising questions about the contributions of malaria control interventions and climate to the transmission trends in South Africa. The shortage of resources, inadequate staff capacity, lack of any new effective intervention tools, and gaps in malaria epidemiology were key concerns, as was the superficially participative nature of the consultation process followed in developing the policy.

## Background

Achievements in the fight against malaria over the past decade are well documented [1]. Despite these gains, malaria continues to be a leading cause of morbidity and mortality in the world, with approximately 214 million annual cases and 438,000 deaths in 2015 [2]. The gains made reflect the decisive steps taken by the international malaria community over the years, including the launch of the Roll Back Malaria (RBM) Movement in 1998, the Abuja Declaration in 2000, the launch of the Eight United Nations (UN) Millennium Development Goals (MDGs) in 2000 and the launch of the Global Malaria Action Plan (GMAP) in 2008 [3,4]. Approximately 663 million clinical cases were averted in endemic areas of Africa between the years 2000 and 2015 [4]. Between 2000 and 2010, the global malaria incidence and deaths decreased by 17% and 26%, respectively [1,5]. In endemic areas of Africa, the incidence of clinical cases decreased by 40% between 2000 and 2015 [4]. Globally, about 109 countries are now free from malaria, but 67 are still malaria endemic with 34 poised to eliminate malaria over the 2013 to 2035 period [5–8].

An increase in international funding, from less than U.S.$ 100 million in 2000 to U.S.$ 1.84 billion in 2012, aided anti-malaria initiatives [9]. Consequently, South Africa, Swaziland, Namibia and Botswana were the first countries declared as ready to target malaria elimination (defined as no local malaria transmission in a defined geographical area) in southern Africa [10,11]. The Lubombo Spatial Development Initiative (LSDI – the cross-border malaria control collaboration between Mozambique, Swaziland and South Africa) has been partly lauded for these reductions [5,12–14]. Malaria cases and deaths in South Africa dropped by 89.4% and 85.4%, respectively, between 2000 and 2010 [15]. Unfortunately, LSDI funding ceased in 2011 [13,15], owing to the global financial difficulties and changes in the Global Fund funding model introduced in 2012 [7]. The launch of MOSASWA (a malaria collaboration between Mozambique, South Africa and Swaziland intended to fill the gap left by LSDI) in July 2015 [16] rekindled hope that the gains made will be protected. However, the U.S.$ 5.1 billion required every year between 2011 and 2020 to achieve universal access to malaria interventions has not been realised, and instead the funding dropped considerably, posing a threat to the effective implementation of the malaria elimination policies in targeted countries [9].

Apart from the global financial difficulties, malaria elimination faces a range of other challenges. For example, a lack of sustained health worker motivation to implement a programme to prevent the reintroduction of cases in Mauritius, was amongst the reasons for the malaria resurgence in 1975 after the country had been declared free of malaria in 1973 [17]. In Ethiopia, shortage of staff, capacity and motivation were identified as key issues likely to threaten the country’s goal of eliminating malaria by 2020 [18]. In Haiti, the meeting of stakeholders comprising laboratory personnel, researchers, clinicians, academics and public health professionals concluded that additional healthcare worker training and the deployment of critical resources were key to achieving the goal of eliminating malaria by 2020 [19]. Sri Lanka, which is now eligible to apply for the WHO (World Health Organization) elimination certification, had its elimination plans in the 1960s frustrated by stakeholders’ complacency, whereby patients with fever were not tested for malaria as a result of physicians’ inertia [5,20]. Furthermore, malaria budgets were cut due to a shift in priorities [20]. Maharaj et al. have raised issues of the malarial parasites’ susceptibility to the drugs of choice, population migration, climatic changes and the evolving complexities concerning malaria epidemiology as potential impediments to achieving the successful elimination of malaria [13].

While studies directly investigating the health providers’ attitudes towards implementing malaria policies are lacking, the foregoing background provides useful insights into malaria elimination globally, and for South Africa, concerning the fight against malaria. It is however by no means the sole precursor of what would make the implementation of malaria elimination in South Africa, or in countries of similar settings, work. Understanding of stakeholders’ knowledge, attitudes, involvement and perceptions about the barriers and facilitating factors for implementing the policy is vital. These are seldom considered. Investigation of the gaps in implementing the user fee exemption policy in health facilities in Ghana, using Lipsky’s theory of street-level bureaucracy [21], found that as frontline workers interact with the people, they make decisions based on professional discretion, culminating in policy modifications, whilst taking note of available resources, costs and practical arrangements. Lipsky’s theory of street-level bureaucracy is defined as public service workers who interact directly with the people targeted by the intervention, and who have considerable discretion during the course of performing their jobs [21,22].

Healthcare workers’ attitude towards the policy has been documented as the key factor affecting the implementation of health policies [22]. Other factors include the lack of engagement of local implementers, the lack of technical consensus, inadequate numbers of human resources, poor leadership, lack of management, and weak health systems [23].

The objective of this study was to investigate the stakeholders’ (malaria researchers, national malaria programme personnel and provincial malaria programme personnel) understanding of the malaria elimination policy in South Africa, the barriers that they perceive and the facilitating factors for effective implementation, since their perspectives in matters relating to the implementing of the elimination policy are of the utmost importance.

## Methods

A total of 12 key informants were purposively selected for their particular interests and participation in malaria elimination policy implementation in South Africa. These included malaria researchers, participants in the provincial malaria programmes in the country’s three malaria endemic provinces (KwaZulu-Natal, Mpumalanga and Limpopo) and participants in the national malaria programme (national Department of Health, Pretoria). They were selected as knowledge producers, policy implementers and policy makers, respectively. The national malaria programme has only three technical staff (all of whom were included in this study) responsible for policy coordination; the rest of the staff are administrative. In the provincial malaria programmes, the managers are the ‘gate-keepers’ of malaria policy implementation, hence their inclusion in this study was considered to be of paramount importance. Selected researchers who were also members of the South African Malaria Elimination Committee (SAMEC – a technical advisory committee guiding the Department of Health on malaria elimination matters) were included. Other authors have raised concerns about policy-making being focussed on policy-makers only but neglecting health researchers [24], hence their inclusion in this study. In fact, long interviews with 2–10 participants are considered to be sufficient to reach saturation in phenomenological studies [25].

Other methods used to identify the key informants were (1) the investigators’ own personal network of contacts in the malaria community, and (2) the researchers’ (key informants) recent contributions to the malaria-related body of knowledge. All 12 key informants agreed to be interviewed (in English) and tape-recorded by the lead investigator. Eight interviews were face-to-face and four were telephonic, owing to the distance and funding challenges. Interviews lasted between 25 and 70 minutes and were based on the semi-structured interview guide adapted in Gase et al. [26]. Interview questions elicited interviewees’ knowledge of the policy and its achievability, including the perceived barriers and facilitating factors to effective implementation (see Supplemental file 1 for full interview schedule).

Audio-tapes were transcribed word for word by an experienced professional transcriber [27]. The first author read all the transcripts while playing the audio-tapes to ensure the accuracy of the transcription and minor corrections were effected. Transcripts were printed and read several times in order to meaningfully organise the data and recognise the emergent themes [28]. Subsequently, the data were manually coded and themes and sub-themes were identified. The individual informants’ stories narrated through their interviews were thematically collated to create a comprehensive picture of their collective views and experiences [27]. The hybrid approach was followed in conducting thematic analysis, meaning that some themes emanated from the interview guide, while others emerged from the data. Basically, the hybrid approach uses a combined technique of inductive and deductive thematic analysis [29].

The study took a constructivist epistemological approach, whereby perception and experience largely informed one’s knowledge [24]. The constructivist worldview is manifest in phenomenological studies in which individuals describe their experiences [30]. Phenomenology is about how actors in a situation experience, conceptualise, perceive and understand a phenomenon [25,31]. It enables the researcher to document research participants’ subjective experiences and interpretations of a particular event, programme or practice [32,33], with a view to obtaining a deeper understanding of how people create meanings of their lived experiences in concrete social situations, thus entering into their inner world [30]. The phenomenological approach used in this study was interpretive phenomenology. While descriptive phenomenology asserts that the researcher needs to shed all his/her prior knowledge, experiences and beliefs about the phenomenon to grasp participants’ essential lived experiences, through what is termed as ‘bracketing’, interpretive phenomenology, on the other hand, values the researcher’s expert knowledge as an insightful guide to making an inquiry meaningful [34].

### Ethical considerations

The study obtained ethics approval (REF: BE240/14) from the University of KwaZulu-Natal Biomedical Research Ethics Committee (BREC). All potential participants received a participant information sheet and an informed consent form prior to their inclusion. All the key informants signed their informed consent prior to being interviewed. All the quotations are anonymised as Key Informants 1–12 in the results section, to protect participants’ identities [23]. Pseudonyms were used in the transcripts which are electronically stored in password-protected files to maintain confidentiality. The hard-copy files are stored in a safe place in the Discipline of Public Health. All data will be kept for five years after the completion of the study.

## Results

The 12 key informants participating in this study were all professionals with varying malaria work experience and duration, ranging from 5 to 37 years (Table 1) with a mean experience of 19 years. Five key informants were females and seven were males. Half [6] were either Ph.D graduates or Professors, one was an M.Sc graduate, two had B.Sc Honours, two had National Diplomas and one had a B.Tech qualification. Six themes and seven subthemes were developed from the analysis.

**Table 1.** Presentation of informants by gender, years of experience in malaria-related work and stakeholder type.

Informants	Gender	Years of experience	Stakeholder type
Key Informant 1	Male	37	Provincial malaria programme
Key Informant 2	Female	5	National malaria programme
Key Informant 3	Female	15	Malaria researcher
Key Informant 4	Male	12	Provincial malaria programme
Key Informant 5	Female	7	National malaria programme
Key Informant 6	Female	20	Malaria researcher
Key Informant 7	Male	31	Provincial malaria programme
Key Informant 8	Male	13	National malaria programme
Key Informant 9	Female	20	Malaria researcher
Key Informant 10	Male	25	Provincial malaria programme
Key Informant 11	Male	24	Malaria researcher
Key Informant 12	Male	12	Provincial malaria programme

### Malaria morbidity, mortality and malaria elimination policy

#### Perceptions towards malaria and malaria elimination policy

**Figure 1.**
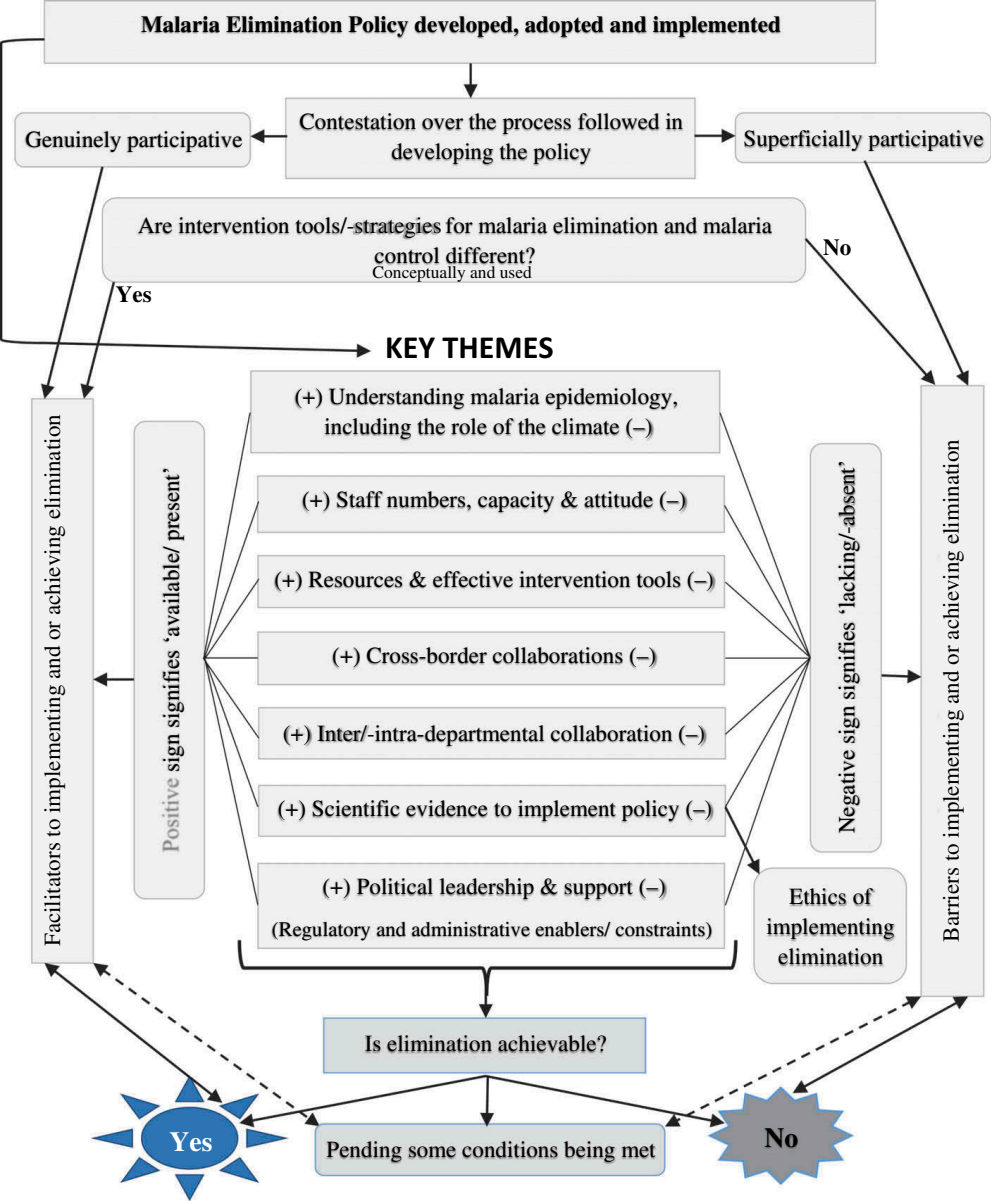
Framework illustrating features affecting the implementation of malaria elimination in South Africa (derived from this study).

The dominant view was that malaria remains a problem in South Africa (Key Informants 1–2, 5–9) and concerns were raised about the recent apparent increase in malaria cases (and deaths) in some endemic areas (Key Informants 2, 5–6, 8) (Figure 1). Some informants dismissed any suggestion that progress has been made since the adoption of the malaria elimination policy in South Africa (Key Informant 4):

From the year 2013/2014 that is where we started to see that there is a bit of an increase in the number of cases or incidence. So, and also looking at the 2014/2015 which is not yet finished – incidence – we have noted that there is a bit of an increase in the incidence rate and that is a cause for concern. If I can just give figures comparing from the last year, if comparing the other year for 2013 and then comparing it with 2014, we actually had 154 deaths which were reported in 2014 which is not good because also going back to the issue of elimination, we have also targeted that by 2015 we would have zero deaths… Compared to 2013 in which we had 105… the previous year 2012, we had 72 deaths. So if you can compare 2012 and 2014 it is more or less like half. It has increased by 50% in terms of the deaths. (Key Informant 2)

Since adoption of malaria elimination policy, things are still the same. I do not see much of a change. (Key Informant 4)

#### Contrasts between control and elimination

There were strong sentiments suggesting that the malaria intervention tools and strategies in South Africa do not reflect a shift from malaria control to malaria elimination (Key Informants 1, 4, 7–8, 10–11). Notably, informants contrasted the two concepts (control and elimination) in respect of the tools each strategy uses, as well as the intended objectives and outcomes:

Spraying is still the mainstay of the programme. Indoor is just basically still the same because we are still using the very same technique of doing [indoor] residual spraying. There is no new technique for elimination. Even health promotion, we are still doing the very same health promotion – same messages we have been sending even on control, we are still sending the same messages even now on elimination. There is [are] no new messages for elimination. (Key Informant 1)

I have read it [policy] through and as I say it annoyed me very much because I could not see that it was an elimination policy. It was a control policy – a modified control policy – bearing in mind that it is not aimed specifically at our vector… because it just repeats exactly what we have been doing in the past 56 years. (Key Informant 7)

#### Achievability prospects of malaria elimination

Three schools of thought emerged with respect to the achievability of the current malaria elimination programme, with the overall impression being that the year 2018 was too soon to be a realistic target (Key Informants 1, 4, 10). The first school thought that malaria can be eliminated in later years, but not in the year 2018 (Key Informants 3, 6). This group was concerned about the timelines set for achieving malaria elimination. The second school of thought was more pessimistic and believed that malaria would never be eliminated in South Africa (Key Informant 7), while the last one believed that elimination was dependent on the adequate deployment of new and effective intervention tools and resources (Key Informants 1, 9–10) (Figure 1). Others attributed the problem to the stakeholders whose mindsets were arguably stuck on control (Key Informants 2–3). These assertions suggest that while malaria case incidence in South Africa meets the WHO threshold for targeting malaria elimination, the intervention tools have rarely been adapted to suit the needs of malaria elimination:

I am worried about the 2018 goal being unrealistic and then disappointing politicians and funders when we do not achieve that goal. So I think we need to be quite careful about how we phrase that message… I do not think we have lot of experience and I think that the few countries in the world that have succeeded with malaria elimination are often vastly different to South Africa. So they are islands, they are deserts, they are very isolated populations where you can imagine much, much less chance of a parasite being reintroduced and often in deserts it is much easier to control vectors. So I do not think any of us has seen very good examples or of places similar to South Africa that have achieved elimination. (Key Informant 6)

And then the other thing that I think that we need to do is to, um, identify which of the planned steps we have not been able to implement like Primaquine and explain to them [politicians] that this is because of a constraint within primarily the Medicines Control Council [MCC] so that he [Minister of Health] does not see it as the failure of the Malaria Control Programme but it is the resources and the system that do not enable us to achieve it. (Key Informant 6)

There is a gross shortage of staff, especially EHPs [environmental health practitioners]. Sometimes there is a team which does not have a team leader and there is a lot of MSAs [malaria surveillance agents] that do not have it [EHP]. There is about 40 vacant posts for MSAs, of which those are very critical posts. We do not have a Lab Manager. We do not have an Entomologist. How can they run this programme without entomologists, without a lab manager, without an HR Manager, without a Financial Manager, without a Systems Manager, so there is a gross shortage of posts, of human resource here? So that is why I am saying before we start talking elimination we should have first levelled the field by filling all these posts. (Key Informant 1)

We would need to move from the mind set of controlling to elimination and also get commitment in terms of funding the programme going forward so that we can achieve elimination. (Key Informant 2)

Other issues affecting the implementation and achievability of malaria elimination were staff capacity and attitudes, intra- and inter-departmental collaboration and stakeholder collaboration (Figure 1). Some informants argued that the closure of the Malaria Research Unit (through organisational restructuring) of the South African Medical Research Council (MRC), which was responsible for managing the country’s malaria information systems, negatively affected the implementation of the malaria elimination strategy (Key Informants 1–2, 8, 11):

So when the Medical Research Council pulled back we were very concerned because then you know it impeded the progress towards implementing the strategy… (Key Informant 8)

Implementing elimination has challenges in that ‘elimination’ does not solely rely on us as workers for malaria control programme. It also relies on other health facilities. Sometimes the doctors and nurses do not report the case in time to us. (Key Informant 1)

And until widespread buy-in is got from the provincial level, even minor acceptance of the policy is virtually non-existent. (Key Informant 3)

#### Cross-border collaborations

Cross-border collaboration was identified as an important influencer on the implementation of the malaria elimination policy in South Africa, including the success prospects (Key Informants 1–2, 6):

It will not help us to do everything this side, whereas at the other side nothing is being done because their effect is going to influence us as well. We should be having meetings now and then with Mozambicans because all of these people who are coming to us, they are coming with the parasite. We have to tell them there is somebody from your area, and the name of the area, maybe they can go there and do something. That is why we need a model like LSDI. We should have something of that sort but [an] extended one. (Key Informant 1)

### Process followed in formulating the malaria elimination policy in South Africa

The view was that malaria cases (Key Informants 1–3, 8), the WHO manuals (Key Informants 2–3, 7–8, 10), the Southern Africa Development Community (SADC) (Key Informants 8, 11), the RBM (Key Informant 3) and other international organisations (Key Informants 3, 10), as well as the local stakeholders (Key Informants 5, 8) were key drivers in the development of the country’s malaria elimination policy. While a few affirmed the local stakeholders’ participation in the process (Key Informants 2, 5, 8), others disagreed (Key Informants 3, 10–11), describing the process as superficial (Key Informant 10) (Figure 1):

It [policy development] was a very intense process because moving towards elimination was not just an idea within the national programme but it involved WHO, it involved programmes that have been there, endemic provinces’ managers and also the non-endemic provincial coordinators which are communicable disease, Centers for Disease Control and Prevention (CDC) coordinators, it involved researchers, it involved experts in malaria not only in case management but health promotion, vector control, so all, even private sector was also involved in terms of engagement for them to be aware that we want to move towards elimination and also not only policy makers in the department, also other interdepartmental coordinators were also involved in terms of drafting that policy. (Key Informant 2)

I think a fundamental error was that this elimination agenda was put together um by the Department of Health without involvement from the people on the ground. So they do not understand where it is coming from and they did not have their input. (Key Informant 3)

There was quite a lot of interaction and we were called quite often to the national offices to discuss and to make inputs but I personally was very frustrated with the process because we will go there and we will raise critical issues and it was just swept off the table. We were a little bit stone-walled. They said, yeah come, come, come, let us talk. Let us develop this policy and there will be a draft on the table and you will spend a whole day… and then three months later the original draft will come back again and I am the one who will ask but what happened to those things I raised, what happened to my comments, what happened to my suggestions? Nothing. So it was a process that continually stuck to the original thing and we never saw our inputs appearing there. (Key Informant 10)

### Malaria epidemiology in South Africa

Knowledge gaps regarding malaria epidemiology in South Africa came under scrutiny, some asserting that weak surveillance systems (Key Informants 3, 10) and misclassification of cases (Key Informant 1) were partly responsible for this gap. A case in point was difficulty in investigating cases among migrants, because such populations often provide misleading residential information to health facilities for fear of victimisation (Key Informants 1, 8), particularly in view of recent xenophobic attacks (Key Informant 6). Secondly, cases emerge sporadically in the least expected areas (Key Informants 7, 10, 12), rendering intervention tools incapable of handling these unanticipated resurgences (Key Informants 1, 6–9). This points to a weak understanding of the malaria epidemiology in the country (Key Informants 7, 10–11), raising questions about the human and non-human (climatic) contributions to these reductions (Key Informant 7) (Figure 1):

The thing we are lacking so much is knowledge. We do not know enough about Arabiensis yet. We need a lot more science. OK, it sounds like passing the buck but, you know, we really do not know enough about this animal. (Key Informant 7)

But most of the cases are coming from Mozambique. But in other countries also, we have some from other countries as well. That is why sometimes we are having those cases which are unclassified because some of these people they do not give leading information to get them where are they. So that is why sometimes we have these unclassified cases. It is because of that. (Key Informant 1)

What I am more concerned about is climate. Malaria is driven principally by the climate. When I see a wet year and cases remain low I will believe we made some progress, but as far as I can see at the moment it is the climate that is controlling malaria. Now that [rain] has to last for at least 10 days for eggs to hatch in order to allow it to erupt as adults [mosquitoes], so in dry periods it does not last that long. There are dust formed puddles, fine, but they do not last long enough to breed mosquitoes. (Key Informant 7)

### Political context of malaria elimination

Four perspectives on the political aspects of malaria elimination emerged, namely: (a) a contested notion of political support to implement the policy (Key Informants 1–2, 6, 8), (b) the development and implementation of malaria elimination policy as a response to a global political mandate/pressure (Key Informants 8, 10), (c) management of cross-border migration and related political sensitivities (Key Informant 6) and (d) the suggestion that the MCC required political influence in making the decisions impacting on the policy implementation (Key Informant 6):

South Africa, the Minister of Health and the Director-General here are very pro and very supportive. So there is a lot of political support and even at the executive level. (Key Informant 8)

I never hear anybody from the political side talking about elimination because, as far as I think by now, we should be having some slots on all TVs and radios talking about elimination. And if maybe our politicians, especially the Minister of Health can take this elimination seriously, and maybe have a lot of slots on different media – it can be TV, radio, newspapers – regular messages coming out to the people, I am sure that can help us a lot. (Key Informant 1)

I think a big political agenda behind it, um and that is also what then happened in the SADC region. There was a decision on malaria elimination taken. South Africa, I think, was to a certain extent politically pressurised to develop a malaria elimination strategy for the country… a few gaps that I have picked up in the entire process of getting the policy approved is that again, to the policy makers and the political head of the Department of Health in South Africa, the full picture was not explained in detail at that level, meaning that yes, a strategy – this strategy is going to cost so much but it is not to say that the strategy will work. (Key Informant 10)

So things like border control, things like, which I think we should not push forward at a time of xenophobia, I think that it is not worth it just to get malaria elimination and start having the horrible war that we used to have during apartheid. (Key Informant 6)

I think that the Medicines Control Council is – there are lots of people trying to improve the efficiency of the system but I think again a clear political mandate saying that this is what the South African government has committed and we need for the Medicines Control Council to not obviously do their job badly but to prioritise the things that will have the most public health impact. So not a bureaucratic inefficiency delaying access to treatment that could release the burden of malaria. (Key Informant 6)

#### State of curatorship and administration in two endemic provinces

Limpopo and Mpumalanga were put under national administration and curatorship in 2011 and 2014, respectively (Key Informants 10, 12), the timing of which adversely affected the implementation of the malaria elimination policy through budget cuts, travel restrictions, a moratorium on the filling of vacant posts, and procurement restrictions:

At the provincial level at the time when the [malaria elimination] policy came in 2011, that was actually a time when Limpopo health was put under national administration. (Key Informant 10)

We are in the curatorship for the whole province, then we cannot buy, we cannot do anything. Like today or this week we were supposed to go to ‘Joburg’ just to get mosquitoes so that we would be able to check the quality of spraying. Now we are stuck because we cannot go there. (Key Informant 12)

### Regulatory constraints and ethics of implementing the policy

#### Regulatory constraints

Two regulatory issues believed to impede the effective implementation of the policy were delays in the registration of Primaquine by the MCC, and non-legislation of malaria elimination as an Act of Parliament as has been done in Mauritius (Key Informants 3, 6, 10):

We haven’t got Primaquine registered which is the best drug to stop onward transmission… (Key Informant 3)

For example, Mauritius created an enabling legislative environment, whereby they passed a Malaria Elimination Act, “a Law of Parliament” that tells the people what they can and cannot do and what they must and must not do. It is an Act of Parliament, it is not just a policy. They are serious about it… (Key Informant 10)

#### Ethics of implementing malaria elimination strategy

A point was made that the elimination policy lacked scientific backing and its implementation may therefore be ethically questionable (Key Informant 10):

If we look at the current South African Malaria Elimination Strategy, I believe there is some activities in there which are not properly informed by scientific evidence and I almost feel it might be almost unethical to implement some of those and the ethics will come into effect that I need to get resources out of the health budget and I might take resources away from another priority programmes if I can fight hard enough for it. I will get resources and spend on an activity that I am not convinced is going to yield the result that we want to get out of it, and to me there is an ethics issue because the money might have been used a little bit better somewhere else in the health system. (Key Informant 10)

### Lessons learned from implementing the policy in South Africa

Informants identified the following lessons to share with countries targeting elimination. Those countries targeting elimination should:

- understand the length, breadth and depth of the situation, using both a desktop exercise and a review of the current malaria situation, to ensure that the strategy is evidence-based before being marketed (Key Informant 8),
- avoid donor dependency and use their own in-country expertise and resources (Key Informants 2, 8),
- embark on comprehensive awareness campaigns while ensuring that there is multi-stakeholder involvement in the elimination agenda (Key Informants 5–6),
- facilitate change management from national to district levels, before establishing a systematic approach to developing and implementing the elimination guidelines (Key Informant 5),
- set realistic and cost-effective goals, drawing from the experiences of successful implementations in comparable settings (Key Informant 6), and
- strengthen surveillance systems to ensure early case reporting, verification and investigation (Key Informant 8).

## Discussion

Consistent with the findings of the paper by Maharaj et al., the results of this study have shown that the achievability of malaria elimination in South Africa remains a subject of intense debate [13]. While there is no denial that over the years malaria morbidity and mortality have been drastically reduced in South Africa [13,15] and elsewhere in the world [1,2], informants shared mixed views as to what led to these reductions. Some claimed that these achievements were as a result of effective malaria control interventions [5,13,35]. Others attributed the successes to changes in the climate, as implicated in other studies [1]. The recent intermittent resurgences of malaria in the least expected areas are not only concerning, but raise questions about the understanding of malaria epidemiology in South Africa. This study revealed that gross shortages of resources and staff capacity and a lack of new effective intervention tools threaten the country’s goal to achieve malaria elimination by 2018. Woyessa et al. made similar observations in Ethiopia, whereby they concluded that the lack of adequately trained healthcare workers and a high attrition rate threatened the country’s goal of eliminating malaria by 2020 [18]. In this study, a concern was raised that ambitious objectives and desired outcomes have been set for South Africa based on intervention tools used previously that have not changed.

The stakeholders’ opinions about the policy were inherently linked to their perceptions about the process followed in its development. Apart from the policy makers, the other informants believed that the process followed in developing the policy was superficially participative. Notably, all participants from the national malaria programme were of the opinion that the nature of the consultations conducted was genuinely and adequately participatory. Others believed though that the key drivers of the policy formulation process were high-level international organisations, such as the WHO and the SADC [10], and South Africa was, to some extent, politically pressured to see the policy through to implementation [11]. Two other politically sensitive issues emerged, namely [1]: the need for strong cross-border collaboration with sensitivities emanating from the recent xenophobic attacks in the country, and [2] the delay in the registration of a transmission-blocking drug called Primaquine. The informants proposed a political intervention to ensure the timeous registration of the drug by the MCC, so that the elimination mandate is not hindered. The feasibility of legislating for malaria elimination, as was done in Mauritius [17], requires further exploratory discussions by the SAMEC members.

Understandably, the implementation would inadvertently be affected by the stakeholders’ perceptions of how the policy was formulated. Studies conducted using Lipsky’s theory of street-level bureaucracy have shown that a lack of genuine participation and full buy-in to a policy development results in frontline healthcare workers making decisions based on their professional discretion, resulting in policy modifications, in taking note of available resources, costs and practical arrangements [21]. In addition, the study conducted by Govere et al. revealed community reluctance to accept malaria interventions as cases decline since they perceive malaria no longer to be a problem [36]. The reportedly poor community consultations during the policy formulation in South Africa may jeopardise community participation in interventions aimed at eliminating malaria. Community involvement in strategy design and implementation proved useful for sustainable malaria elimination on the Vanuatu Islands [37].

In contrasting malaria control and malaria elimination, one informant suggested that the elimination policy was actually riding on the achievements of the malaria control interventions and the current dry climate. The modelling study conducted by Caminade et al. has previously implicated climate in malaria, but was unable to establish direct causality between the climate and malaria distribution trends [1]. One informant in this study explained that water bodies lasting long enough for mosquitoes to develop into full adults is crucial and this is supported by the literature [6]. The suggestion by some respondents that the reduction in malaria transmission in South Africa is mainly due to the changes in climate, rather than to the recent interventions, requires further investigation to better understand how the climate has affected the epidemiology of malaria, so that interventions can be better adapted to the context. Regardless of the climate’s impact on malaria reduction, there remains a need for understanding the malaria epidemiology in South Africa. Settings with low malaria transmission are known to experience evolving complexity and challenges regarding malaria epidemiology [5]. Knowledge about the epidemiology of malaria in countries seeking to eliminate the disease is an important pillar for continual improvements of targeted interventions [5] and SAMEC members need to have frank discussions concerning these issues.

Irrespective of the diversity of opinions concerning malaria elimination and the epidemiology thereof, the year 2018 was overwhelmingly seen as too soon to eliminate malaria in South Africa. Similar sentiments have been echoed by other authors [38,39]. The suddenly increasing numbers of malaria cases and deaths, at a time when the gains need to be consolidated, are concerning. However, this phenomenon is not unique to South Africa, as neighbouring Botswana experienced outbreaks in the Okavango district in 2013 and 2014, soon after celebrating significant malaria incidence reduction [40]. The central question about whether malaria elimination is achievable or not received mixed views. While few were optimistic about elimination, some were completely pessimistic and others believed that malaria elimination was subject to certain conditions being met [13].

Deep concerns were raised about the contribution of population migration to the rising number of malaria cases. Cotter et al. asserted that imported malaria cases should be addressed to achieve malaria elimination [5], as they were the reasons for resurgences in the elimination setting of Zanzibar [14]. Effective control of imported malaria cases requires strong cross-border collaboration with the neighbouring malaria endemic countries. Swaziland is going through similar experiences with endemic neighbouring Mozambique [12,41]. The LSDI, which ended in 2011, was lauded for its successes in drastically reducing malaria in collaborating countries and hopefully, the newly established MOSASWA will be well equipped to address the malaria importation challenges in the respective countries. Weak collaborations are occurring not only at cross-border level, but at intra- and inter-sectoral levels as well, typified by reports of poor partnership between malaria programmes and health facilities, doctors and nurses. The closure of the Malaria Research Unit of the MRC appears to have adversely affected the implementation of the elimination policy.

There is documented evidence suggesting that challenges faced by eliminating countries are likely to render the current traditional intervention tools insufficient to free countries from the last few cases [5]. Key amongst these are the human movements [40]. In fact, one of the most important shortcomings of the Global Malaria Eradication Programme of 1955–1969 was the dependence on single interventions and the failure to change control measures as the malaria situation evolved [11]. This raises questions about whether the current interventions are in keeping with how the malaria situation has evolved in South Africa. The feeling that malaria elimination is not sufficiently topical in South Africa is concerning. The ethical aspects raised by one informant, regarding the implementation of this contentious policy, should not be ignored. Countries planning to embark on elimination should draw from the lessons raised by the informants in this study.

### Strengths and limitations of the study

The most important strength of this study was its ability to capture and analyse the three key types of stakeholders’ perspectives on the implementation of malaria elimination in South Africa in a single study, thus revealing the converging and diverging nuances within and between stakeholders, with respect to facilitators and barriers to policy implementation. The study limitations included that (a) it was conducted on a national scale with limited resources, thus constraining the performance of member checks and conducting interviews and analysis iteratively to ensure redundancy and saturation; and (b) the research process followed a linear pattern, which began with conducting all interviews, followed by the translation and lastly data analysis, thus denying the true value of the iterative nature of qualitative data analysis [28]. Despite these constraints, the study produced insightful and useful findings.

## Conclusions

The majority of informants were in agreement that the year 2018 was too soon for malaria elimination in South Africa. While some were optimistic about the achievability of malaria elimination in South Africa, others were not. Most concerns raised pertained to the lack of new tools and resources and the inadequate understanding of malaria epidemiology. Three schools of thought emerged with respect to what was required to achieve malaria elimination in South Africa, namely (a) new intervention tools, (b) additional resources, and (c) a complete overhaul of the system guided by research-informed intervention tools and malaria epidemiology. Irrespective of the viewpoints and the complexities thereof, this policy has certainly created interest in scrutinising health systems’ capacity to transition South Africa to being a malaria-free country in a manner not previously seen. This paper anticipates bringing into the public domain the debates on malaria elimination issues. Such issues have hardly been debated openly for fear of contributors being labelled as ‘pessimists’. These results would also be useful for other countries with similar settings, aiming to eliminate malaria.

## Supplementary Material

Supplemental Data

## Paper context

Global achievements in malaria reduction through various interventions are well documented. The call to target malaria elimination in countries with low malaria transmission has particularly been based on promising evidence about malaria’s decline. Apart from a wide body of knowledge in areas such as laboratory science, insecticide and drug susceptibility, and vector behaviour, studies investigating stakeholders’ perceptions towards the policy implementation in eliminating countries are lacking.
