# Supplementary material for: Towards the implementation of malaria elimination policy in South Africa: the stakeholders’ perspectives
Source: Glob Health Action. 2017 May 5;10(1):1288954. doi: 10.1080/16549716.2017.1288954 (PMC5496171; doi:10.1080/16549716.2017.1288954)
Supplement: Supplemental Data [file ZGHA_A_1288954_SM6453.pdf]

Title: Factors influencing the implementation of the malaria elimination policy in South Africa

Interview Questions Asked of Key Informants

Category of Questions	Key Informant Category		
	**Researchers**	**Malaria Personnel**	**Policy Makers**
**Interviewee details**	1. Age in last birthday<br>2. Sex<br>3. Nationality<br>4. Employing organisation<br>5. Country of work/ employment<br>6. Current job designation/ title<br>7. Highest qualification<br>8. Years of experience in current job	1. Age in last birthday<br>2. Sex<br>3. Home province (nationality if not South African)<br>4. Work province<br>5. Current job designation/ title<br>6. Highest qualification<br>7. Years of experience in current job	1. Age in last birthday<br>2. Sex<br>3. Current job designation/ title<br>4. Highest qualification<br>5. Years of experience in current job
**Knowledge, attitudes and perceptions concerning malaria elimination**	9. From research point of view, to what extent is malaria elimination agenda a priority for you? Elaborate<br>10. Are you concerned about the level of malaria prevalence in South Africa? Why/ why not?<br>11. In your opinion, do you think it is feasible to achieve malaria elimination in South Africa by 2018? Elaborate<br>12. Who do you think should be involved in implementing malaria elimination policy (at	8. To what extent is malaria elimination agenda a priority to you? Elaborate<br>9. Are you concerned about the level of malaria prevalence in your operational setting? Why/ why not?<br>(a) How is it being controlled<br>(b) Are cases increasing, decreasing or staying the same?<br>(c) Is malaria still a problem?	6. To what extent is malaria elimination agenda a priority to you? Elaborate<br>7. Are you concerned about the level of malaria prevalence in South Africa? Why/ why not?<br>(a) How is it being controlled<br>(b) Are cases increasing, decreasing or staying the same?<br>(c) Is malaria still a problem?<br>(d) Since the adoption of malaria elimination policy, what has been achieved?

	individual/ group/ or organizational level)?<br>13. How familiar are you with the South African malaria elimination policy? Elaborate<br>14. Do you know what process was followed in developing this policy? Elaborate	(d) Since the adoption of malaria elimination policy, what has been achieved?<br>10. Do you think it is feasible to achieve malaria elimination in South Africa by 2018? Elaborate<br>11. Who do you think should be involved in implementing malaria elimination policy (at individual/ group/ or organizational level)?<br>12. How familiar are you with the South African malaria elimination policy? Elaborate<br>13. Do you know what process was followed in developing this policy? Elaborate	8. Who should be involved in implementing malaria elimination policy in South Africa? Elaborate<br>9. What will it take to achieve elimination by 2018?<br>10. Who did the process of developing the malaria elimination policy involve and why?
**Distinguishing features between malaria control and elimination**	15. Please share key similarities between malaria control strategy and malaria elimination (if any)<br>16. Please share key differences between malaria control strategy and elimination (if any)	14. Please share key similarities between malaria control strategy and malaria elimination (if any)<br>15. Please share key differences between malaria control strategy and elimination (if any)	11. What are the key distinguishing features between South Africa's malaria control policy of 2007 and the current malaria elimination policy?
**Barriers and facilitating factors to implementing malaria elimination policy**	17. Have you observed or experienced any barriers to implementing malaria elimination policy? Elaborate<br>18. Do you forsee any opportunities to enhance the current malaria elimination policy? Elaborate<br>19. How feasible do you think it is to implement malaria elimination policy? Elaborate	16. Have you experienced any barriers to implementing malaria elimination policy? Elaborate<br>17. Do you forsee any opportunities to enhance the current malaria elimination policy? Elaborate<br>18. How feasible it is to implement malaria elimination policy? Elaborate	12. To date, have you experienced or observed any barriers to implementing malaria elimination policy? Elaborate<br>13. Do you forsee any opportunities to enhance the current malaria elimination policy? Elaborate<br>14. If you were to share with another country targeting elimination the key lessons you have learned so far with regards to

	20. How might malaria elimination policy affect your organization and your work?<br>21. How easy or difficult do you think it is to manage and enforce this policy?<br>22. What barriers might be faced in implementing malaria elimination policy or strategy? How could these barriers be overcome?	19. How does malaria elimination policy affect your Department and your work? Elaborate<br>20. How easy or difficult it is to implement this policy?<br>21. What are the barriers to implementing this policy or strategy? How could these barriers be overcome?	implementing malaria elimination policy, what would they be?
**General questions**	23. If you were to be entrusted with the overall strategic responsibility to ensure that malaria elimination is achieved in South Africa:<br>a. Is there anything new you would introduce? Elaborate.<br>b. Is there anything you would withdraw/ cancel? Elaborate.<br>c. Is there anything you would retain as is or improve on? Elaborate.<br>24. Is there anything else you want to share pertaining to the implementation of malaria elimination policy in South Africa?	22. If you were to be entrusted with the overall strategic responsibility to ensure that malaria elimination is achieved in South Africa:<br>a. Is there anything new you would introduce? Elaborate<br>b. Is there anything you would withdraw/ cancel? Elaborate<br>c. Is there anything you would retain as is or improve on? elaborate<br>23. Is there anything else you want to share pertaining to the implementation of malaria elimination policy in South Africa?	15. Is there anything else you want to share pertaining to the implementation of malaria elimination policy in South Africa?

Adapted in Gase et al. 2011
